# Anti-Sulfoglucuronosyl Paragloboside Antibody: A Potential Serologic Marker of Amyotrophic Lateral Sclerosis

**DOI:** 10.1177/1759091416669619

**Published:** 2016-09-28

**Authors:** Dongpei Li, Seigo Usuki, Brandy Quarles, Michael H. Rivner, Toshio Ariga, Robert K. Yu

**Affiliations:** 1 Department of Neuroscience and Regenerative Medicine, Medical College of Georgia, Augusta University, GA, USA; 2 Laboratory of Biomembrane and Biofunctional Chemistry, Faculty of Advanced Life Science, Frontier Research Center for Advanced Material and Life Science, Hokkaido University, Sapporo, Japan; 3 Department of Neurology, ALS Clinic, Medical College of Georgia, Augusta University, GA, USA

**Keywords:** amyotrophic lateral sclerosis, sulfoglucuronosyl paragloboside, ganglioside, anti-glycolipid antibody, Functional Rating Scale, affinity parametric complex criterion

## Abstract

Amyotrophic lateral sclerosis (ALS) is a neurodegenerative disease characterized by progressive degeneration of upper and lower motor neurons. Although the etiology of ALS is obscure, genetic studies of familiar ALS suggest a multifactorial etiology for this condition. Similarly, there probably are multiple causes for sporadic ALS. Autoimmune-mediated motor neuron dysfunction is one proposed etiology for sporadic ALS. In the present study, anti-glycolipid antibodies including GM1, GD1b, GD3, and sulfoglucuronosyl paragloboside (SGPG) were investigated in the sera of a large number of patient samples, including 113 ALS patients and 50 healthy controls, by means of enzyme-linked immunosorbent assay with affinity parametric complex criterion evaluation and thin-layer chromatography immunooverlay (immuno-TLC). Anti-SGPG antibodies were found in the sera of 13.3% ALS patients (15 out of 113). The highest titer reached 1:1600. The presence of anti-SGPG antibodies in the serum samples was also confirmed by immuno-TLC. Importantly, a multiple logistic regression analysis showed that the presence of anti-SGPG antibody was positively correlated with age (*p* < .01) and negatively correlated with ALS Functional Rating Scale score (*p* < .05). Moreover, the localization of SGPG-immunoreactivity on the motor neurons of rat spinal cord and a mouse motor neuronal cell line, NSC-34 was observed by an immunofluorescence method. These data suggest that SGPG could represent a specific pathogenic antigen in those ALS patients. The presence of anti-SGPG antibodies in the serum of ALS patients should represent a diagnostic biomarker of ALS, and it could reflect the severity of the disease.

## Introduction

Amyotrophic lateral sclerosis (ALS) is a progressive neurologic disorder causing loss of motor neurons in the motor cortex, brain stem, and spinal cord, resulting in atrophy and weakness of skeletal muscles along with corticospinal tract dysfunction (Sreedharan, 2010; Garbuzova-Davis and Sanberg, 2014). Most ALS cases are sporadic, but 5% to 10% of cases are genetically linked familial ALS. More than 100 ALS-associated genes have been identified (Peters et al., 2015), including a genetic mutation in Cu/Zn superoxide dismutase (Rosen et al., 1993; Siddique and Deng, 1996). Although the etiology and pathogenic mechanisms of sporadic ALS are obscure, there is considerable evidence that there might be multiple etiologies producing ALS. An autoimmune mechanism may be one such mechanism responsible for the initiation and progression of this disease (Barbeito et al., 2010; McCombe and Henderson, 2011; Malaspina et al., 2015). Auto-antibodies against calcium channels and certain anti-glycolipid antibodies have been reported in sera of certain ALS patients (Engelhardt et al., 1995; Smith et al., 1996; Niebroj-Dobosz et al., 1999). However, the significance of these antibodies in ALS has not been well elucidated.

Sulfoglucuronosyl paragloboside (SGPG) is one of the glycolipid target antigens in various peripheral neuropathies (Leger et al., 1996). Anti-SGPG antibody has been reported by us as well as other groups to be present in the serum samples of some ALS patients (Maeda et al., 1991; Ben Younes-Chennoufi et al., 1995; Ikeda et al., 2000). However, its involvement in the neuropathogenesis of ALS has not been studied in a large number of patients with well-documented clinical and demographic records, which precludes its clinical significance. For this reason, we deemed that a detailed study on the occurrence of anti-SGPG was warranted in a clearly defined patient population for functional correlation.

SGPG is also located in the microvessels of adult rat brain, which suggests the reaction of circulating anti-SGPG antibodies with SGPG on the microvessels may potentially cause the breakdown of the blood–brain barrier or blood–nerve barrier, resulting in the penetration of these antibodies to target the antigenic sites on the nervous tissues for degeneration (Yu et al., 1990). Since ALS is a motor neuron disease, the localization of SGPG on motor neurons is of particular significance, as it imparts a cellular specificity for degeneration in this disease. For this reason, we are particularly interested in the occurrence SGPG antibodies and its potential relationship to ALS.

In the present study, anti-glycolipid antibodies including GM1, GD1b, GD3, and SGPG were investigated in the sera of 113 ALS patients as well as 50 healthy controls by means of an enzyme-linked immunosorbent assay (ELISA) with affinity parametric complex criterion (APCC) evaluation. The presence of these anti-glycolipid antibodies in serum samples was confirmed by immuno-thin-layer chromatography (immuno-TLC). The relationships of age, gender, race, clinic symptoms, forced vital capacity (FVC), as well as Functional Rating Scale (ALSFRS) scores with the presence of anti-glycolipid antibodies, were also assessed. An immunofluorescence method was used to confirm the localization of SGPG-immunoreactivity on the motor neurons of rat spinal cord and a mouse motor neuron cell line, NSC-34, which is a hybrid cell line produced by fusion of motor neuron enriched, embryonic mouse spinal cord cells with mouse neuroblastoma (Cashman et al., 1992).

## Methods

### Study Subjects

One hundred and thirteen (113) ALS patient serum samples received from the ALS Clinic at the Medical College of Georgia, Augusta University, and 50 healthy male and nonpregnant female subjects were enrolled in the study. All ALS patients were examined in the AUHealth ALS clinic and met the El-Escorial criteria for possible, probable, or definite ALS. Demographic characteristics such as age, gender, and race as well as clinical symptoms, FVC and ALSFRS scores were collected.

### Antibody Measurement

The presence of anti-glycolipid (GM1, GD1b, GD3, and SGPG) antibodies was first screened by means of ELISA. We used 25 ng each of GM1, GD1b, GD3, and SGPG, dissolved in methanol, as the antigens to coat the wells of the ELISA plate. The solvent in each well was dried in air. Nonspecific bindings were blocked by incubation of each well with 1% bovine serum albumin in phosphate-buffered saline (PBS) for 30 min. Serum samples were diluted 1:400 and added in duplicate onto the plate followed by incubation at room temperature overnight. Commercial normal human serum (Jackson ImmunoResearch, West Grove, PA) was treated as control. Horseradish peroxidase-conjugated anti-human IgG or IgM was used as the secondary antibody. After addition of the secondary antibody, the plate was developed with o-phenylenediamine dihydrochloride (Sigma-Aldrich, St. Louis, MO) peroxidase substrate solution. The optical density (OD) value of each well was measured at 492 nm with a microplate spectrophotometer.

The P/N ratio was calculated by dividing the OD value of test serum by the OD value of the normal control. Samples with a P/N ratio > 2.1 were regarded as positive.

Positive samples were then further tested by serial dilutions (1:200, 1:400, 1:800, 1:1600, and 1:3200). Since we had frequently encountered serum samples that presented high background OD values, the serially diluted serum samples were compared using glycolipid-coated and noncoated ELISA plate wells to avoid the high background values which could be misinterpreted as positives (so-called *false positives*). Anti-glycolipid antibody titers were assigned as the highest dilution at which the adjusted OD value (OD value of glycolipid-coated well minus OD value of noncoated well) was ≧0.1. Specific antibody binding activity was evaluated by APCC value calculated according to our previous report (Usuki et al., 2014). Briefly, affinity parametric complex 1 was calculated using diluted serum data by glycolipid-coated wells of an ELISA plate and was given as a formula composed of three-extrapolated parameters: hill slope (H1), subtraction absorbance value of top minus bottom (T1 − B1), and diluting factor (D1), where 50% of the antibody exhibits the response (D50). $\text{APC}1=(\text{T}1-\text{B}1{)}^{1/\text{H}1}{\text{D}1}^{1/\text{H}1}$. APC2 is calculated using ELISA serum dilution data from control well in the same plate. $\text{APC}2=(\text{T}2-\text{B}2{)}^{1/\text{H}2}{\text{D}2}^{1/\text{H}2}$.

$$\text{APCC}=\frac{(\text{T}1-\text{B}1{)}^{1/\text{H}1}{\text{D}1}^{1/\text{H}1}}{(\text{T}2-\text{B}2{)}^{1/\text{H}2}{\text{D}2}^{1/\text{H}2}}$$

### Confirmation by Thin-Layer Chromatography Immunooverlay

The presence of anti-glycolipid antibodies in the serum samples was also confirmed by immuno-TLC. Briefly, 300 ng of an antigen (GM1, GD1b, GD3, and SGPG) was spotted on each lane of a TLC plate. The TLC plate was then developed with the solvent of chloroform: methanol: 0.25% CaCl_2_ (55:45:10, by volume). After the plate was dried in air, it was dipped in 0.25% poly-isobutyl-methyl acrylate in n-hexane, which was used as the coating agent. Nonspecific bindings were blocked by incubation with 1% bovine serum albumin in PBS for 30 min. The TLC plate was incubated with the serum sample (1:50) overnight. Horseradish peroxidase-conjugated anti-human IgG or IgM was used as the secondary antibody enhanced chemiluminescent blotting kit (Thermo Fisher Scientific, Waltham, MA) for X-ray film was used for revealing the positive bands.

### Immunofluorescence Staining

NSC-34 cells were cultured on poly-D-ornithine-coated glass coverslips in Dulbecco's Modified Eagle's Medium (DMEM) plus 10% fetal bovine serum for 3 days, and then the fetal bovine serum was removed for further differentiation. Cells were fixed in 4% paraformaldehyde in PBS for 30 min and stored in PBS at 4℃ for immunofluorescence staining.

Normal rats were anesthetized by intramuscular injection of ketamine hydrochloride (40 mg/kg body weight) and xylazine hydrochloride (4 mg/kg body weight), then perfused with 4% paraformaldehyde in PBS. After perfusion, lumbar spinal cords were removed and postfixed for 4 hr at 4℃ in the same 4% paraformaldehyde solution. The blocks were then equilibrated in sucrose (30% in PBS) and sectioned (35 µm).

Cells and sections were washed three times in PBS and incubated in 1% bovine serum albumin (containing 1% Triton X-100 in PBS) for 30 min prior to overnight incubation at 4℃ with the primary monoclonal antibody specific for the sulfoglucuronosyl epitope of SGPG (mAb NGR50; Yamawaki et al., 1996). After rinsing in PBS for 3 × 10 min, the sections were incubated in corresponding Alexa Fluor 568 goat anti-mouse IgG (Invitrogen, 1: 2000) at room temperature for 2 hr. After washing, the slide was coverslipped in 0.01 M PBS containing 50% glycerol, then examined under a Leica fluorescence microscope. We used 1% bovine serum albumin instead of the primary antibody as a negative control, and the results were negative.

### Statistical Analysis

Statistical analyses were performed using the SPSS 16.0 version software program. The association between age, gender, race, clinic symptoms, FVC, as well as ALSFRS score and the presence of anti-glycolipid antibody were evaluated using multiple logistic regression analysis. A *p* value of .05 was considered to be statistically significant. Best-curve fitting was performed by nonlinear regression analysis of GraphPad Prism 5.0 software package (GraphPad, San Diego, CA).

## Results

**Figure 1.**
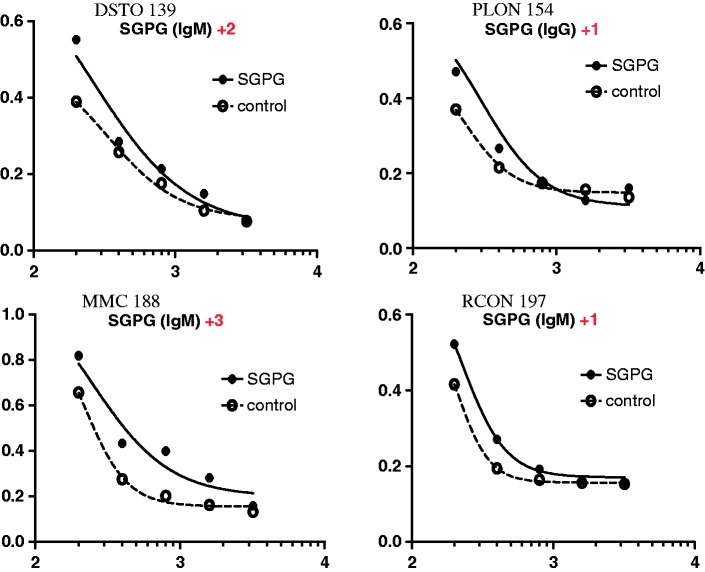
Representative best-fit curves for serum dilution equation (APC1 and APC2) using the enzyme-linked immunosorbent assay data from four anti-SGPG antibody positive samples. X-axis represents dilution (10^x^) and Y-axis represents absorbance at 492 nm. Data points from SGPG-coated wells are shown as closed circle (•) on solid lines, and those from control-coated wells are shown as open circle (○) on dotted curves.

Demographic characteristics of the subjects recruited in the present study are shown in Table 1. The ELISA results showed that 15 out of 113 (13.3%) tested ALS subjects showed varying degrees of positive responses to different antigens. The remaining samples had practically no activity against the test antigens. It is interesting that all of these 15 samples showed positive anti-SGPG IgG or IgM antibody activities. Seven of them also showed anti-GM1 IgG or IgM activities, six showed anti-GD3 IgG or IgM activities, and four showed anti-GD1b IgG or IgM activities. Upon serial dilution, all 15 samples showed positive but varying degree of anti-SGPG antibody titers, with the highest titer being at 1:1600. However, only 3 out of 50 (6.0%) healthy control samples showed positive anti-SGPG IgG or IgM antibody activities. A summary of anti-glycolipid antibody evaluation using the APCC method is shown in Table 2. Figure 1 shows four representative best-fit curves for serum dilution equation using the ELISA data from anti-SGPG antibody positive samples.

**Table 1.** Demographic Characteristics of the Subjects Recruited in the Study.

Groups	Healthy controls (*N* = 50)	ALS patients (*N* = 113)
Characteristics	Values expressed as %	
Age
16–64	60.0	56.6
≥65	40.0	43.4
Gender
Male	26.0	54.0
Female	74.0	46.0
Race
Caucasian	92.0	77.9
African American	8.0	22.1

*Note.* ALS = amyotrophic lateral sclerosis.

**Table 2.** Summary of Antibody Evaluation Using APCC.

	SGPG		GD3		GD1b		GM1	
	IgG	IgM	IgG	IgM	IgG	IgM	IgG	IgM
DSTO 139	–	+2	–	–	–	–	–	–
LJAC 139	+1	+1	–	–	–	–	–	+2
PLON 154	+1	–	+2	–	+1	–	+1	–
RSHA 158	+2	+1	+1	–	–	–	–	–
TCAN 184	+1	+1	–	–	–	–	–	–
LNOR 185	+1	+1	–	–	–	–	–	–
MMC 188	–	+3	–	+1	–	–	–	+3
TLDN 191	–	+2	–	–	–	–	–	–
RCON 197	–	+1	–	–	–	–	–	–
KGOO 212	+1	+1	–	–	+1	–	+1	–
CGAR 228	+1	+1	–	+1	–	–	–	+1
DSAV 229	–	+2	–	–	–	+2	–	–
CHOL 230	–	+3	–	–	–	+3	–	–
SCOL 238	+1	–	+1	–	–	–	+1	–
MHOU 240	+1	+1	–	+1	–	–	–	+1

*Note.* SGPG = sulfoglucuronosyl paragloboside. Serum samples with APCC < 1.5 (−),1.5 < APCC < 3.0 (+1), 3.0 < APCC < 5.0 (+2), and APCC > 5.0 (+3).

**Figure 2.**
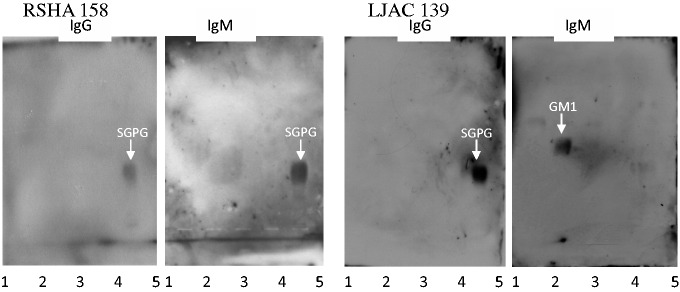
Immuno-thin-layer chromatography confirmation of the presence of anti-glycolipid antibodies in two representative positive samples. Lane 1: Human brain ganglioside mixture; 2: GM1; 3: GD1b; 4: GD3; 5: SGPG. The developing solvent system was chloroform: methanol: 0.25% CaCl_2_ (55:45:10, by volume). In panel 4, lane 1, the small amount of GM1 in human brain ganglioside mixture was too low to be detected.

The presence of anti-SGPG IgG or IgM activities was confirmed in the positive samples by means of immuno-TLC. Anti-GM1 IgM was also confirmed similarly in one of the samples (Figure 2).

In the multiple logistic regression analysis, the presence of anti-SGPG antibody served as the dependent variable and demographic characteristics, clinical symptoms, FVC as well as ALSFRS scores were entered as independent variables. The results showed that the presence of anti-SGPG antibody was positively correlated with age (*p* < .01) and negatively correlated with ALSFRS score (*p* < .05). Gender, race, FCV level, and other clinical symptoms such as tongue atrophy and fasiculations as well as other bulbar-related symptoms were not significantly related to the presence of anti-SGPG antibody (Table 3).

**Table 3.** Multiple Regression Using the Presence of Anti-SGPG Antibody as a Dependent Variable.

Parameters	Chi-square	Regression coefficient	*SE*	*p*	Odds ratios [95% CI]
Age	9.937	0.079	0.027	.004^**^	1.082 [1.026, 1.141]
FVC	1.230	0.017	0.015	.275	1.017 [0.987, 1.048]
ALSFRS	4.540	−0.108	0.054	.047*	0.898 [0.807, 0.999]
Gender	0.441	0.467	0.709	.510	1.595 [0.398, 6.396]
Race	0.428	−0.518	0.782	.508	0.596 [0.129, 2.759]
Tongue symptom	0.314	0.439	0.789	.578	0.578 [0.331, 7.282]
Bulbar symptom	0.728	0.707	0.844	.402	0.402 [0.388, 10.603]

*Note.* FVC = forced vital capacity; ALSFRS = amyotrophic lateral sclerosis Functional Rating Scale.

* *p* < .05. **p < .01.

**Figure 3.**
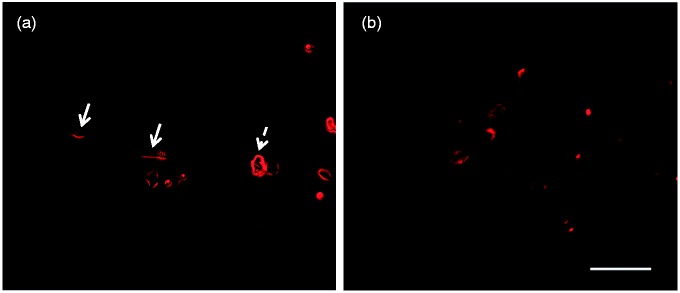
(a) Sulfoglucuronosyl paragloboside immuno-positive activities on NSC-34 cells. Dashed arrow marks the undifferentiated hybridoma cells, and solid arrow marks the cell bodies of differentiated motor neurons. (b) SGPG-positive motor neurons within the anterior horn of rat spinal cord. Bar = 100 µm.

To identify the presence of SGPG in motor neurons, immunofluorescence staining was carried out to detect the localization of SGPG-immunoreactivity in NSC-34 cells and rat spinal cord. In NSC-34 cells, the SGPG positive immunoreactivity was found on the surface of undifferentiated hybridoma cells and differentiated motor neurons, especially on the surface of cell bodies. In rat spinal cord sections, the SGPG positive reactivity mainly existed on the motor neurons of anterior horn. Based on the size of those positive cells, most of them are likely alpha (α) motor neurons (Figure 3). Due to the lack of freshly autopsied human spinal cords, we did not perform a similar study on human motor neuron samples.

## Discussion

In the present study, anti-SGPG antibodies were found in the sera of 13.3% ALS patients (15 out of 113) by means of ELISA. The presence of anti-SGPG antibodies in the serum samples was also confirmed by immuno-TLC. Age and ALSFRS scores were found for the first time to be correlated with the presence of anti-SGPG antibodies. Most interestingly, we also showed that the localization of SGPG on the motor neurons of NSC-34 cells as well as rat spinal cord, consistent with the notion that these glycolipids may represent the target antigens underlying the pathogenic mechanisms of motor neuron disease.

The identification of anti-SGPG antibodies in a significant proportion (13.3%) of a large patient population with ALS is noteworthy and consistent with our previous studies (Maeda et al., 1991). But the positive rate is relatively lower compared with previous data. This might be due to the self-serum nonglycolipid coated controls used in the present ELISA study. Since high-background OD values were frequently encountered in glycolipid ELISA tests of human serum samples, self-serum nonglycolipid coated controls were performed in the present ELISA study to avoid false positive results caused by the dark background arising from of the serum. Moreover, we carried out serial dilutions of the samples and confirmed the authenticity of our data using the APCC method (Usuki et al., 2014).

It should be mentioned that SGPG and its homolog, sulfoglucuronosyl lactosaminyl paragloboside have been reported to be present in cancer cells of neuroectodermal origin; anti-SGPG antibodies have also been detected in sera of patients with metastatic neural tumors but not in healthy controls (Ariga et al., 2008). This may be because HNK-1-bearing glycoconjugates, including glycolipids, are upregulated and exposed on the tumor cell surface. However, compare to other neurological disease, such as Guillain-Barré syndrome, the percentage of positive anti-SGPG antibody in ALS patients is much higher than that in other neurological disorders (our unpublished data).

Anti-GM1 antibodies were also found in 6.2% (7 out of 113) of the ALS samples, but the positive rate was much lower than those in other neurological diseases, such as Guillain-Barré syndrome (Simone et al., 1993; Kaida et al., 2009). Therefore, the presence of anti-SGPG antibodies in the serum of ALS patients is unique.

It is interesting that the presence of anti-SGPG antibody was correlated with age and negatively correlated ALSFRS score. The ALSFRS provides an estimate of the patient’s degree of functional impairment (“The Amyotrophic Lateral Sclerosis Functional Rating Scale,” 1996). The present statistic results suggest that the presence of anti-SGPG antibody might reflect the severity of the disease.

ALS is associated with the selective loss of motor neurons in the motor cortex, spinal cord, and brain stem. The presence of anti-SGPG antibodies in ALS patient serum suggests that motor neurons may bear specific target antigen(s), such as SGPG and sulfoglucuronosyl lactosaminyl paragloboside, responsible for the immune attack. Therefore, it is necessary to detect the presence of SGPG on motor neurons. Since the human spinal cord sample is unavailable at the moment, we performed immunofluorescence staining to detect the localization of SGPG in NSC-34 cells and rat spinal cord. The results confirmed that the motor neurons do contain SGPG. Interestingly, most of the immunopositivity appears to be associated with α motor neurons. For this reason, the presence of anti-SGPG antibodies in the serum of this subgroup of ALS patients may initiate motor neuron degeneration by targeting SGPG on α motor neurons.

To study the pathogenic role of SGPG in ALS, we have carried a preliminary study by sensitizing Lewis rats with SGPG. All rats sensitized with SGPG developed high titers of anti-SGPG antibodies, and 50% animals developed mild to moderate neurological symptoms, such as distal tail tone loss (Maeda et al., 1991). However, except for damage to the endothelial cells in the spinal cord, which suggested a breakdown of the blood–brain barrier; and axonal change found in the dorsal columns of spinal cord, which suggested proprioception loss could not explain the neurological symptoms in the experimental animals. Further studies are needed to confirm the effects of serum anti-SGPG antibodies.

SGPG was reported to react with anti-ELEC-39, HNK-1, and NC-1 antibodies as well as anti-voltage-gated calcium channel antibodies (Mailly et al., 1989; Appel et al., 1993). It is suggested that these protein antigens in the nervous tissues could bear the SGPG/HNK-1 carbohydrate epitope and react with anti-SGPG antibody, thereby involve in the pathogenesis of ALS.

In conclusion, our present study suggests that SGPG and/or an sulfoglucuronosyl epitope bearing molecule is a target antigen in a significant number of patients diagnosed as having ALS. We can safely conclude that anti-SGPG antibody can be used as a serum biomarker of ALS, and this antibody may be useful for presymptomatic diagnosis and therapeutic monitoring of new therapeutic strategies.

## Author Note

The nomenclature for gangliosides is based on that of Svennerholm (1963) and IUPAC-IUBMB Joint Commission on Biochemical Nomenclature (“The nomenclature of lipids. Recommendations (1976) IUPAC-IUB Commission on Biochemical Nomenclature”, 1977).
